# Extreme thrombocytosis with severe anemia and infection in a Sudanese patient: A case report

**DOI:** 10.1016/j.amsu.2022.104927

**Published:** 2022-11-17

**Authors:** Zainab Alghali Elsaid Muhammed, Mohammed Alfatih, Ashraf Saeed Hussein Babiker, Khadeega Mohammed Ahmed Aldau, Mona babiker alattaya mohamed, Hamza Elsiddig Hajali, Maysam ELsayed Saeed Gharbawi, Mohannad Abdalfdeel Almahie Shaban, Abdellatif Ahmed

**Affiliations:** a Shendi University Faculty of Medicine and Surgery, Sudan; b Alzaiem Alazhari University Faculty of Medicine, Sudan; c University of El Fasher, Sudan; d Martyr Ali Abdel Fattah Hospital, Sudan; e Sudan Medical Specialization Board, Sudan; f National University - Sudan Faculty of Medicine, Sudan

**Keywords:** Reactive thrombocytosis, Iron deficiency anemia, Sudanese, Infection, GSC, Glasgow Coma Scale, HCT, Hematocrit, PID, Pelvic Inflammatory disease

## Abstract

**Introduction:**

Secondary thrombocytosis, also known as reactive thrombocytosis, is defined as an abnormal increase in platelet count as a result of another underlying medical or surgical condition. Once the medical cause of reactive thrombocytosis was determined, it could be treated. In this case, supportive treatment with no iron supplements for anemia and infection improved the case condition rapidly.

**Case presentation:**

we report a 20 years old Sudanese female who presented with high-grade fever, right iliac fossa pain, hyper pigmented macules on the tongue and a past history of undiagnosed anemia. Laboratory results showed platelets = 1007 × 10^3/μl, hemoglobin = 3.5 g/dl with low MCV, total WBC was also high = 14.9 × 10^3/μl. Peripheral blood picture showed anisocytosis and poikilocytosis, microcytic hypochromic RBCs associated with target cells, pencil cells, teardrops cells and polychromies cells and with leukocytosis and very high platelets in the film. Abdominal ultrasound showed evidence of pelvic inflammatory disease. After receiving supportive treatment, antibiotics and 3 units of blood the patient showed remarkable improvement and reduction in platelet count.

**Discussion:**

We discuss the mechanism of the reactive thrombocytosis state and the variable treatment options when accompanied with iron deficiency anemia.

**Conclusion:**

Reactive thrombocytosis with extreme platelet count should always be considered in patients presented with severe iron deficiency anemia and infection. In this case report the high platelet count was reversed successfully after commencing antibiotics and blood transfusion although of the poor patient compliance and the poor investigations were obtained from the patient.

## Highlights

• The Extreme thrombocytosis state with severe iron deficiency anemia and infection could be treated with supportive treatment that doesn't include iron supplements.
• A very high platelet count should always be considered in patients presented with severe anemia and infection.
• The close follow up for hemoglobin level and the platelet count in secondary thrombocytosis could prevent the fatal complications.

## 1 Introduction

Unlike essential thrombocytosis which is unregulated increase production of platelet in the bone marrow, secondary thrombocytosis or better known as Reactive thrombocytosis is defined as an abnormal increase in platelet count as a consequence of other underlying medical or surgical condition, or as a side effect of the usage of certain drugs. Reactive thrombocytosis is accounted for 80%–90% of thrombocytosis cases and is often discovered in routine laboratory results [1].

Secondary thrombocytosis may occur in acute settings such as in acute blood loss or acute infections, or may accompany chronic medical illnesses like cancer, chronic infections, asplenia, or iron deficiency anemia. After addressing the underlying cause of reactive thrombocytosis the return of platelet count to its normal limit is expected [2]. Secondary thrombocytosis is usually an asymptomatic reaction but extreme thrombocytosis may lead to thrombotic events such as acute myocardial infarction, mesenteric vein thrombosis, and pulmonary embolism [3].

## 2 Patient information

Our patient is a 20 years old Sudanese female who was admitted to our medicine department in Martyr Ali Abdel Fattah Hospital on June 2022 with a chief complaint of high-grade fever and right iliac fossa pain radiating to the back for 1 day. There was no associated nausea, vomiting, change in bowel habits or weight loss.

Personal history revealed that the patient had pica symptoms in the last two years as she consumed a large amount of ice on a daily basis. She had normal menstrual cycle history for the past two years. She had no family history of chronic diseases or anemia. Her social history was unremarkable for smoking, alcohol drinking and she was not sexually active.

## 3 Examination

Examination revealed a fully conscious patient (GCS = 15), pulse was 110 beats per minute, and blood pressure was 80/60 mmHg.

**Fig. 1:**
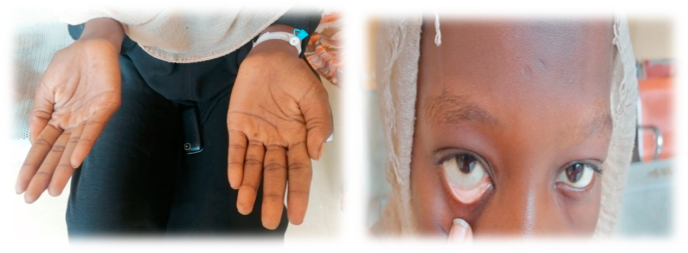
Shows pallor of the skin and conjunctiva.

**Fig. 2:**
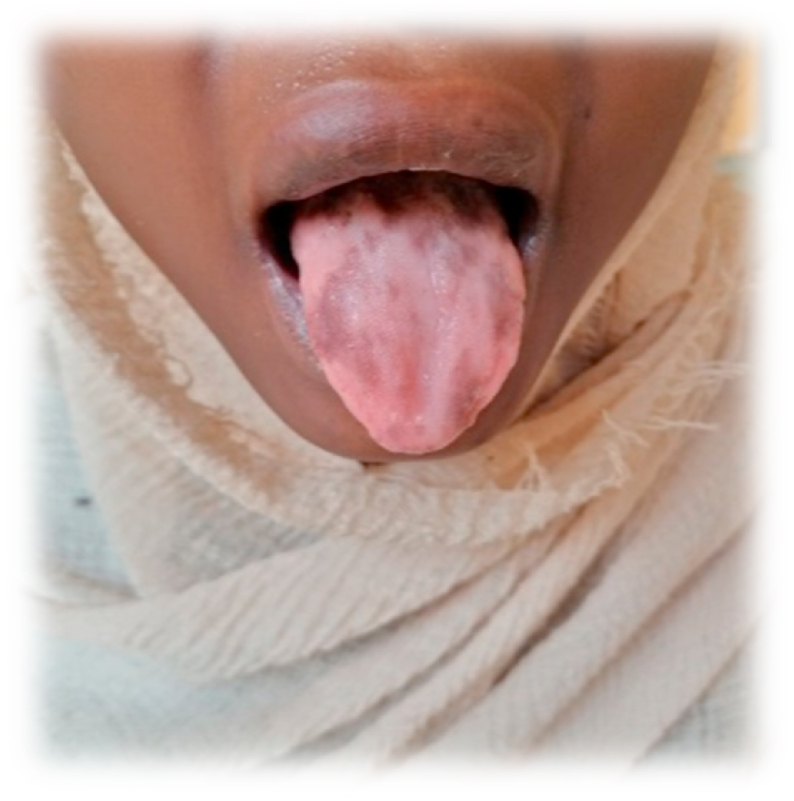
Shows angular stomatitis, atrophic glossitis and multiple brown hyperpigmented macules on the dorsal surface of the tongue. (For interpretation of the references to colour in this figure legend, the reader is referred to the Web version of this article.)

Upon examining her skin and mucus membrane, skin and conjunctival was pallor (Fig. 1). Atrophic glossitis and angular stomatitis were noted also (Fig. 2). There was no oropharyngeal erythema or any signs of inflammation. The oral mucosa and gingiva were normal without lesions.

Her Cardiac examinations revealed normal first and second heart sounds. When examining her abdomen, her liver and spleen were not palpable; the only finding was a pain in the right iliac fossa without rigidity or rebound tenderness. The patient was also examined by the surgical department to rule out the acute abdomen.

## 4 Investigations

Initial laboratory workshop for her revealed anemia with hemoglobin = 3.5 g/dl, HCT = 12.4%, mean corpuscular volume = 55.1fl, platelets = 1007 × 10^3/μl which is very high and total WBC was also high (14.9 × 10^3/μl) with Neutrophils about 80%. The renal function test plus electrolytes, and liver function test were all normal.

The abdominal ultrasound showed features of Pelvic inflammatory disease. It showed mild to a moderate collection of fluid in the posterior cul-de-sac and multiple tiny calculi near her right kidney. NO PID swaps were obtained due to the patient refusal.

Peripheral blood picture shows anisocytosis and poikilocytosis, microcytic hypochromic RBCs associated with target cells, pencil cells, teardrops cells and polychromies cells and with leukocytosis and very high platelets in the film. Based on the presence of low hemoglobin, HCT, low MCV, high platelet count and findings on the peripheral blood picture, iron studies and bone marrow biopsy were requested.

Unfortunately, iron parameter tests and bone marrow biopsy were not available in the hospital and the patient refused to be referred to another hospital to do these tests.

## 5 Treatment and follow-up

With regard to the treatment this patient received, it was mainly supportive in nature since we did not confirm the diagnosis this patient may have. The patient was hypotensive and immediately received two Normal saline units of 0.9% 500 ml infusion in the emergency room and was requested to do the routine investigations.

On-ward patient immediately received Ceftriaxone 1 gm IV and was requested to do peripheral blood picture which revealed anisopoikilocytosis and severe microcytic hypochromic cells.

For the second, third and fourth day the patient received 3 units of blood. She continued to have Ceftriaxone 1 gm IV injections and potassium citrate powder. The patient remarkably improved but refused to stay at the hospital and was discharged on the 4th day. Her platelet count was followed on the 5th day which showed improvement to (720 × 10^3/μl).

The patient was followed up in the referred clinic for a period of 12 days with no significant complications and another platelet count followed 8 days after discharge which was normal (260 × 10^3/μl).

Finally, the patient was requested to do iron studies and a bone marrow biopsy but she neglected to continue the investigations. No further workup was done as the patient's symptoms and conditions improved rapidly and the patient refused to come to the follow-up.

## 6 Discussion

The mechanisms are causing the reactive thrombocytosis state in iron deficiency anemia are unknown. There are several reports that explained the mechanisms of reactive thrombocytosis from the aspect of thrombopoietic cytokines. One of these was Akan et al. study which demonstrated the role of the serum levels of thrombopoietin, erythropoietin, leukemia inhibitory factor, IL-6, and IL-11 and it found that none of these cytokines had any effect on reactive thrombocytosis in iron deficiency anemia [4].

Also in a case report of V. Uzel et al. about severe thrombocytosis in iron deficiency anemic 12 years old girl concluded that the cause of thrombocytosis in iron deficiency is not fully understood and it is very important that children should be evaluated immediately for infection and iron deficiency before performing further examinations [5].

The clinical examination for this patient suggests the presence of anemia (the severe pallor in the hands and conjunctiva). Also, the hyperpigmentation in her tongue is suggesting the presence of Pigmented fungiform papilla because there is no associated skin, nail, or other cutaneous changes.

The pigmented fungiform papilla is a normal variant of the tongue that has no associated pathologic significance. This finding usually presents in late childhood and does not change over time [6]. More common in patients with dark skin but may be found in any race.

The characteristics and colour of the pigmentation vary and the most common presentation is diffuse patches or macules on the dorsal surface of the tongue. The patches may be seen on the anterior and lateral surface of the tongue or at the tip of the tongue. The colour variation of these patches may range from brown to dark black [7].

Regarding the treatment for such a case treating the reactive thrombocytosis caused by iron deficiency with iron supplements has shown to be very effective and would rapidly correct the platelet count. In a case report by Kristin Bergmann et al. of 34 old woman who had undergone bariatric surgery 5 years previously, a diagnosis of reactive thrombocytosis due to iron deficiency secondary to iron malabsorption was made. Their finding emphasizes the importance of regular control of the possible need for iron supplementation following bariatric surgery [8]. The iron supplements also made a significant response to the treatment in the case report of arterial and venous thrombosis caused by reactive thrombocytosis and iron deficiency anemia in Deepak Venugopalan Pathiyil et al. study [9].

In this case, the patient refused to do iron studies and bone marrow biopsy. She was offered counselling many times on how these investigations would help with the management. Therefore, No iron supplements were prescribed to the patient. She only agreed to have a short course of antibiotics (Ceftriaxone IV injection) and 3 units of blood.

The patient improved quickly after the treatment commenced. The platelet count rapidly decreased on the 5th day after admission and returned to normal level on the 12th day.

## 7 Conclusion

Reactive thrombocytosis should always be considered in patients present with severe iron deficiency anemia and infection. This case showed that in the absence of iron supplements therapy, the reactive thrombocytosis state for this patient improved quickly by only treating the infection and the anemia. The hemoglobin level and platelet count should be followed closely in patients with reactive thrombocytosis to prevent fatal complications of anemia. Iron studies and bone marrow biopsy are mandatory to rule out any bone marrow malignancies and to reach a definitive diagnosis.

## Ethical approval

The ethical approval not required.

## Source of funding

This study was self-funded.

## Author contribution

Zainab Alghali Elsaid Muhammed conceived the idea, set the study design and collected the data. Ashraf Saeed Hussein Babiker, Mohammed Alfatih and Maysam ELsayed Saeed Gharbawi took full detailed history, did Examinations and Investigations. Zainab Alghali Elsaid Muhammed wrote the first draft. It was critically revised by Abdellatif Ahmed and Hamza Elsiddig Hajali. Khadeega Mohammed Ahmed Aldau, Mona babiker alattaya mohamed, Mohannad Abdalfdeel Almahie Shaban performed the literature review.

## Consent

Written informed consent was obtained from the patient for publication of this case report and any accompanying images. A copy of the written consent is available for review by the editor-in-chief of this journal.

## Registration of Research Studies

1 Name of the registry: Not required
2 Unique Identifying number or registration ID: Not applicable
3 Hyperlink to your specific registration (must be publicly accessible and will be checked)

## Guarantor

Zainab Alghali Elsaid Muhammed. Shendi University Faculty of Medicine, Khartoum, Sudan. zooobaalghali@gmail.com. ORCID: 0000-0002-8089-079X

## Provenance and peer review

Not commissioned, externally peer-reviewed.

## Declaration of competing interest

Authors report no conflict of interest.
